# A genome-wide scan for signatures of differential artificial selection in ten cattle breeds

**DOI:** 10.1186/1471-2164-14-908

**Published:** 2013-12-21

**Authors:** Sophie Rothammer, Doris Seichter, Martin Förster, Ivica Medugorac

**Affiliations:** 1 Chair of Animal Genetics and Husbandry, Ludwig-Maximilians-University Munich, Veterinärstr. 13, 80539 Munich , Germany; 2 Tierzuchtforschung e.V. München, Senator-Gerauer-Straße 23, 85586 Grub, Germany

**Keywords:** Selection signature, XP-EHH, Cattle, Artificial selection, Adaptation

## Abstract

**Background:**

Since the times of domestication, cattle have been continually shaped by the influence of humans. Relatively recent history, including breed formation and the still enduring enormous improvement of economically important traits, is expected to have left distinctive footprints of selection within the genome. The purpose of this study was to map genome-wide selection signatures in ten cattle breeds and thus improve the understanding of the genome response to strong artificial selection and support the identification of the underlying genetic variants of favoured phenotypes. We analysed 47,651 single nucleotide polymorphisms (SNP) using Cross Population Extended Haplotype Homozygosity (XP-EHH).

**Results:**

We set the significance thresholds using the maximum XP-EHH values of two essentially artificially unselected breeds and found up to 229 selection signatures per breed. Through a confirmation process we verified selection for three distinct phenotypes typical for one breed (polledness in Galloway, double muscling in Blanc-Bleu Belge and red coat colour in Red Holstein cattle). Moreover, we detected six genes strongly associated with known QTL for beef or dairy traits (*TG, ABCG2, DGAT1, GH1, GHR* and the Casein Cluster) within selection signatures of at least one breed. A literature search for genes lying in outstanding signatures revealed further promising candidate genes. However, in concordance with previous genome-wide studies, we also detected a substantial number of signatures without any yet known gene content.

**Conclusions:**

These results show the power of XP-EHH analyses in cattle to discover promising candidate genes and raise the hope of identifying phenotypically important variants in the near future. The finding of plausible functional candidates in some short signatures supports this hope. For instance, *MAP2K6* is the only annotated gene of two signatures detected in Galloway and Gelbvieh cattle and is already known to be associated with carcass weight, back fat thickness and marbling score in Korean beef cattle. Based on the confirmation process and literature search we deduce that XP-EHH is able to uncover numerous artificial selection targets in subpopulations of domesticated animals.

## Background

Since the Neolithic Transition humans have systematically made use of many different production capabilities (work, meat, milk, fibre, etc.) associated with various domesticated animal species. For cattle, the domestication of the aurochs took place about 10,000 years ago in two isolated events, one in the region of the Fertile Crescent, the other in the Indus Valley [1,2]. During the time of divergent selection into the approximate 953 cattle breeds that currently exist locally [3] striking phenotypical differences concerning, among other things, size, coloration, behaviour and performance have occurred. Finding links between phenotypical and genotypical changes is of great importance in order to ascertain a better understanding of genetic adaptation and presents the opportunity to improve breeding work through directed selection on favourable genotypes.

In general, recent technological and analytical advances of genomics hold great promise for the future when it comes to moving from hypothesis-driven candidate gene studies to hypothesis-generating genome-wide scans in various species [4]. Essentially, there are two different ways to generate hypotheses for relationships between genotype and phenotype. On the one hand, classical association studies use distinct phenotypes to design mapping populations for which the genomes are scanned. On the other hand, it is possible to first scan the genome and detect regions under natural or artificial selection, so called selection signatures, and then to identify the corresponding selected phenotype and adaptive pathways. Since the availability of cost-effective high-throughput genotyping methods, a number of studies have been performed with the aim of detecting genome-wide signatures of selection in cattle. These studies used different methods for the identification of “classical selective sweeps”, i.e. the processes in which beneficial mutations arise and rapidly increase in frequency while simultaneously reducing or eliminating the variation of linked neutral sites [5,6]. There are diverse patterns of variation caused by strong positive selection on beneficial mutation and various statistical tests exploit this distinct information concerning selective sweeps [7]. Based on the statistical tests applied, the recent genome-wide mappings of selective sweeps in cattle can be divided into three groups: (i) exploiting high-frequency of derived alleles by Fay and Wu’s *H* Test [8] (performed by MacEachern *et al.*[9]), (ii) population differentiation using diverse methods based on allele frequency differences [9-15], and (iii) test for long haplotypes applying either EHH [16], iHS [17] or Rsb [18] (performed by: EHH [19], iHS [12,13,20], Rsb [20]). The advantage of detecting long haplotypes is that these represent the most recent signatures of selection [21] and should therefore harbour changes in the genome caused by the latest selective pressures including breed formation and performance gain.

Some results from the human 1,000 Genomes Project indicate that classical selective sweeps are rare in recent human evolution but in fact the selection on standing variation is more common [22]. Innan and Kim [23] performed simulation studies that suggested selection on standing variations is even more probable in domesticated species than in natural outbreed populations. However, particular consideration was given to strong artificial selection in a domestication event itself. The genetic architecture of domesticated cattle, similar to other domesticated species, is not only shaped by intensive selection during domestication but also by very recent strong selection in a population artificially fragmentised into breeds and by the usage of modern reproduction techniques (artificial insemination, embryo transfer, sperm sorting) for assortative mating in these effectively very small subpopulations (e.g. [24]). As per the definition, modern breeds are reproductively isolated and the appropriate breeding associations maintain these artificial barriers. During the foundation of the breeds some breed specific characteristics were fixed and others amplified based on limited local genetic variation [25]. Therefore, the specific genetic architecture of domesticated populations should be accounted for rather than the debatable tendency to automatically project almost all assessments from human genetics into domesticated species [24].

During the “classical selective sweep” a typical signature of selection arises around the selected or so-called “core”-alleles [5,6]. These core-alleles thus combine two characteristics: (i) high frequency, which is typical for old alleles that have already had much time to rise in frequency, and (ii) at the same time a remarkable length of the haplotype surrounding it, typical for young alleles. To detect these signatures of selection Sabeti *et al.* developed the Extended Haplotype Homozygosity (EHH; [16]). EHH is defined as the probability that two randomly chosen haplotypes carrying the same core-allele are homozygous for the entire interval from the core to a given locus. Voight *et al.* introduced a further development called integrated Haplotype Score (iHS) based on the ratio of the integrated EHH-curves of the two (ancestral and derived) core-alleles [17]. Cross-Population Extended Haplotype Homozygosity (XP-EHH; [26]) is essentially based on both EHH and iHS, with the main difference that it is calculated between and not within subpopulations. Here the EHH-curves are calculated and integrated for each of the two subpopulations (not alleles) separately. XP-EHH is then calculated as the ratio of those subpopulation-specific integrals, so there is no need to distinguish between ancestral or derived alleles as for iHS.

This study explores the adaptive genetic variation fixed or concentrated within artificially sub-divided and divergently selected breeds. The between-population scan for signatures of strong recent artificial selection was accomplished by applying Sabeti’s XP-EHH [26] to genome-wide SNP data (47,651 SNPs) of ten divergent cattle breeds. The chosen breeds are either highly selected for milk or beef or represent dual purpose breeds (milk and beef) as well as virtually unselected cattle strains without defined breeding goals or official breeding organisations. Furthermore, we perform a principal component analysis of these ten breeds, estimate pairwise *F*_*ST*_-values as well as the effective population size of these breeds, discuss the consequences of applying different concepts to selection signature studies and provide some perspectives on future strategies for identifying the selected variants in an artificially sub-divided population.

## Methods

### Ethical statement

No extra sampling was applied for this study, thus no formal ethical approval was required. Instead, we used hair roots, blood and semen samples that are collected by breeding associations and insemination stations on a regular basis. They were as follows: for Anatolian Black, Illyrian Mountain Buša, Red Holstein, Blanc-Bleu Belge and Galloway and some individuals from German Fleckvieh, Murnau-Werdenfelser and Franken Gelbvieh we used existing DNA from a previous study, which had already been prepared [27]. For other individuals of Murnau-Werdenfelser, DNA was extracted from hair roots or blood samples collected by the respective breeding association for regular quality control of breeding records, namely paternity testing. The required blood sampling is conducted by certified veterinarians who follow the German Animal Welfare Act which avoids unnecessary pain, suffering and damage to the animals. Breeders collected hair root samples directly by plucking hair from the pinna or the tail. DNA preparation of samples from Original Braunvieh and Braunvieh and the remaining individuals of the German Fleckvieh, Franken Gelbvieh and Murnau-Werdenfelser breeds was done from semen samples collected by approved commercial artificial insemination stations as part of their regular husbandry procedures in the cattle industry.

### Animal samples

Samples from more than 1,800 animals representing ten distinct cattle breeds were collected from 1992 to 2008. The sampled breeds can be divided into four breeding-purpose groups. The Anatolian Black (ABB) and the Illyrian Mountain Buša (IMB) are local varieties of an initially large Buša cattle population kept in almost the entire Balkan region and parts of Turkey [27]. Since there is no official breeding organization and even no common breeding goal, we provisionally consider these two breeds as “artificially unselected” [25,28,29]. The cattle breeds Original Braunvieh (OBV), Murnau-Werdenfelser (MWF), German Fleckvieh (DFV) and Franken Gelbvieh (FGV) represent dual-purpose breeds of the alpine regions typically bred for dairy and beef traits with parallel intensity. The remaining four breeds are specialized for dairy (Red Holstein (RH) and Braunvieh (BBV)) and beef production (Galloway (GLW) and Blanc-Bleu Belge (BBB)). According to the geographic origin BBB, RH and GLW represent the north-western, BBV, DFV, FGV, MWF and OBV the alpine, and IMB and ABB the south-eastern regions of Europe. BBV originated from the intensive upgrading of OBV by American Brown Swiss [25,28]. The origin of samples and number of sampled individuals per breed are listed in Table 1.

**Table 1:** Collected breeds and samples

**Breed**	**Code**	**Purpose**	**Origin**	***Ng*** **( *Na *)**	**mUAR**	**aUAR**^**d)**^
Anatolian Black	ABB	Artificially unselected	Turkey	48 (43)	0.045	-0.024
Illyrian Mountain Buša	IMB	Artificially unselected	Albania	52 (43)	0.177	-0.024
German Fleckvieh	DFV	Dairy-beef	South Germany	723 (50)	0.099	-0.020
Original Braunvieh	OBV	Dairy-beef	Switzerland	48 (35)	0.191	-0.029
Murnau-Werdenfelser	MWF	Dairy-beef	South Germany	106 (46)	0.199	-0.021
Franken Gelbvieh	FGV	Dairy-beef	Central Germany	139 (50)	0.156	-0.020
Braunvieh ^a)^	BBV	Dairy	South Germany	568 (50)	0.077	-0.020
Red Holstein	RH	Dairy	North Germany	62 (50)	0.101	-0.020
Blanc-Bleu Belge	BBB	Beef	Belgium	47 (31)	0.148	-0.032
Galloway	GLW	Beef	Scotland ^b)^	47 (32)	0.160	-0.033
Sum	10	4	3 ^c)^	1840 (430)		

Breed name and code, breeding purpose, geographic origin of breed (Origin), number of samples genotyped (Ng) and number of samples used for selection signature analyses (Na) as well as maximum UAR (mUAR) and average UAR (aUAR) for each breed.

^a)^German Braunvieh upgraded by US American Brown-Swiss.

^b)^Originating from Scotland but sampled in Germany.

^c)^Three geographic origins representing the north-western, the alpine, and the south-eastern regions of Europe.

^d)^Negative relationships are caused by taking the current generation as base population and cannot be interpreted as probabilities but as the correlation of homologous alleles in different gametes. (for details see [35]).

### Genotyping data and quality control

DNA was genotyped with Illumina BovineSNP50 BeadChip [30] using standard procedures [31]. This SNP-Chip contains 54,001 SNPs with a mean distance of 48.78 kb. Markers that met one of the following criteria were excluded from further analysis: (i) unknown position according to the reference assembly of the bovine genome UMD3.1 [32,33], (ii) minor allele frequency (MAF) across all 430 independent (see below) animals of less than 0.0023, (iii) frequent paternity conflicts in reference animals with known paternity or (iv) marker call-rate of less than 0.90. After filtering, 47,651 SNPs remained for further analysis.

### Reconstruction of haplotypes

Haplotypes were reconstructed by a Hidden Markov Model implemented in the program *BEAGLE 3.0.4*[34]. Since additional animals and pedigree information may further improve the accuracy of haplotype reconstruction, we extended the haplotyping design by 2,032 animals otherwise not relevant for this study. Finally, the haplotyping-process was based on 1,572 parent-offspring pairs, 92 parent-offspring trios and 2,008 unrelated individuals.

### Unified additive relationships analysis

Differences in the degree of familial relationships within analysed breeds may have an impact on the distribution of haplotypes and allele frequencies and could therefore lead to biased results. To sample pure bred and most independent animals we used pedigree records collected by breeding associations where possible. For the two “artificially unselected” cattle breeds (ABB and IMB) there were no breeding associations and no pedigree records available. Instead, we used written and oral evidences from animal owners to ensure the largest possible independence of the sampled animals. To ensure a similar population structure within each breed, regardless of pedigree availability, we estimated genome-wide unified additive relationships (UAR; [35]) between individuals. Within an iterative procedure, we excluded the animal with the highest UAR in each iteration until the highest relationship to one or more animals within the same breed was below 0.20. In order to illustrate the efficiency of the chosen limit, we did not preselect the DFV individuals based on pedigree but took more than 700 DFV bulls representing the most important breeding bulls of more than two generations. From this relatively large number of bulls only 55 animals remained after the iterative UAR procedure. This shows that the chosen limit of UAR < 0.20 is a reasonable compromise between largest possible sample-independence and sufficient final sample size. According to simulation studies the XP-EHH procedure maintains its power to detect selection signatures with as few as 20 chromosomes representing the population under study [36]. To ensure the analyzed DFV animals and the individuals of the remaining nine breeds were of similar age, we excluded the five oldest DFV bulls. Moreover, to avoid population stratification problems we only used unrelated OBV animals originating from Switzerland, BBB animals originating from Belgium and only the black strain of GLW. After population stratification (BBB, GLW and OBV) and familial relationships correction (UAR analysis of all breeds) 430 animals remained for further analysis with XP-EHH (*Na*, Table 1).

### Principal component analysis and estimation of pair-wise fixation index

To ensure we didn’t have population stratification within the sets of animals and to be able to assess the genetic diversity of the ten breeds, we conducted a principal component analysis (PCA) and estimated the pair-wise fixation index (*F*_*ST*_) based on Weir and Cockerham [37] using R packages *pcaMethods*[38] and *hierfstat*[39]. For both procedures the input data were the genome-wide genotypes of all 47,651 markers coded as “-1” (homozygosity allele 1), “0” (heterozygosity) and “1” (homozygosity allele 2) for PCA and “11” (homozygosity allele 1), “12” (heterozygosity) and “22” (homozygosity allele 2) for *F*_*ST*_-calculation.

### Application of the XP-EHH test

To estimate XP-EHH values we applied the software implementation by Pickrell *et al.*[36]. The criterion for XP-EHH value estimation was relaxed so that estimates for regions with greater marker distances could be obtained (kindly provided by Dr. Joseph Pickrell). This adapts the program to the lower marker density of our data compared to human SNP data.

For the estimation of unstandardised XP-EHH values each of the 47,651 SNPs was automatically treated as “core-SNP” once. This was done for all ten breeds in comparison with each of the nine remaining. The unstandardised XP-EHH values of each breed comparison were then standardised (sXPEHH) separately so as to have zero mean and unit variance. For each pair of breeds, the breed, which was searched for selection signatures, was called “case breed” and the one used as reference population “control breed”. Due to the absence of genomic position we instead used the physical position (1 Mb ≈ 1 cM).

### Identification of chromosomal regions under strong artificial selection

To identify chromosomal regions under selection we used the “artificially unselected” Buša breeds, ABB and IMB, to define the significance thresholds of the sXPEHH values. Since these breeds are not consciously selected in the sense of modern breeding, the length and frequency of their haplotypes should be mainly due to genetic drift, which generally reflects the effective population size (*N*_*e*_), and an inevitable selection for overall fitness (pressure of more ‘natural’ environments; [27]). In contrast, in systematically selected breeds kept in favourable environments (environmental pressures are managed through interventional husbandry strategies) a beneficial allele affecting a breed specific or an economically important trait will be under strong artificial selection; a much more directional force than the two mentioned above. Here we defined chromosome-wide significance thresholds for each control breed separately. Therefore, we determined the maximum sXPEHH value of the case breed IMB (maxIMB) and the maximum sXPEHH value of the case breed ABB (maxABB), both with respect to a specific control breed. These values depict the limits that breeds, without targeted selection, are able to reach if compared to the respective control breed. The assumption is that chromosomal regions under strong artificial selection are able to cross these limits. Since there is also some selection within IMB and ABB we defined the significance threshold as the average of maxIMB and maxABB. All SNPs of any case breed are therefore qualified as “significant” if they exceed the threshold defined for the specific control breed. This procedure is illustrated in (Additional file 1: Figure S1). Moreover, all significance thresholds defined for each control breed-chromosome combination are listed in (Additional file 2: Table S1).

We were primarily interested in breed specific selection signatures confirmed by multiple evidences. Therefore, we merged all significant SNPs of a case breed regardless of the respective control breeds. For each breed, we integrated all significant SNPs to a common signature, if separated by one non-significant SNP at the most. Moreover, the significant signatures included half of the physical distance to the neighbouring non-significant marker on both sides (example given in Additional file 3: Figure S2). Nevertheless, some significant SNPs were still isolated. Since XP-EHH in general searches for unexpected long haplotypes, we only considered these isolated significant SNPs (“singletons”), if confirmed in comparison to more than one control breed. Such singletons were otherwise disregarded from further examinations; a filtering step only feasible due to our multi-breed design.

All genes lying within defined signatures were considered as positional candidate genes.

### Estimation of the effective population size

The effective population size (*N*_*e*_) was estimated based on its known relationship with linkage disequilibrium (*LD*) and the inter-marker genetic distance *c*[40] as described in Flury *et al.*[41]. For this purpose, we excluded all monomorphic SNPs and those with a minor allele frequency below 0.10 and estimated the pair-wise *r*^*2*^-values using Haploview [42]. We then grouped the *r*^*2*^-values over all 29 autosomes in distance bins with an increment of 50 kb starting the first bin at 975 kb and ending the last at 10,025 kb for each breed separately. The bin-midpoints therefore varied from 1,000 kb (~0.01 Morgan) to 10,000 kb (~0.10 Morgan). For the estimation of *N*_*e*_ the physical distance was used as an approximation of the genetic distance *c* (1 Mb = 0.01 Morgan). Since *N*_*e*_ is estimated (2c)^-1^ generations ago [43], these bins consequently represent *N*_*e*_ from 50 to 5 generations ago. For each bin we calculated the mean of the *r*^*2*^-values E(*r*^*2*^) and finally estimated *N*_*e*_ based on the equation

$$N_{e}=\frac{{\left(E\left(r^{2}\right)-1/n\right)}^{-1}-1}{4c}$$

where *n* is twice the number of analysed animals (*Na*, Table 1) of the respective breed.

## Results

### Principal component analysis and fixation index

**Figure 1:**
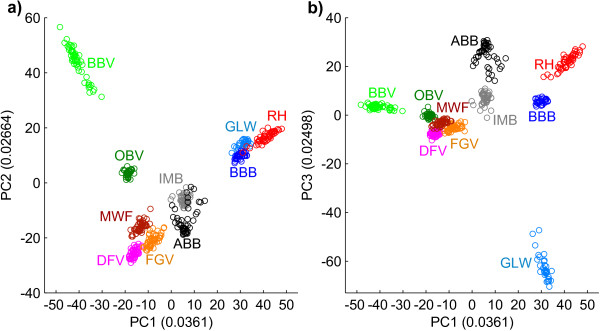
**Principal component analysis based on 47,651 genotypes.** For all 10 breeds principal components (PC) and their importance (numbers in brackets) are shown. **a)** PC1 vs. PC2 **b)** PC1 vs. PC3.

As shown in Figure 1, only the first principal component (PC1) was needed to divide the ten breeds according to their geographic origin. The alpine breeds (DFV, MWF, FGV, OBV, BBV) and the north-western breeds (GLW, BBB, RH) are located at opposite sites, whereas the breeds ABB and IMB are located in the middle of these groups. PC2 and PC3 permit an accurate distinguishing of ABB and IMB and the north-western breeds (GLW, BBB, RH). In contrast, for the alpine breeds MWF, FGV and DFV there are more components needed for a definite separation.

The pair-wise *F*_*ST*_-values are listed in Table 2. The highest differentiation was found between BBV and GLW. All other breeds also showed the highest differentiation either with BBV (north-western breeds) or GLW (alpine and artificially unselected breeds). The highest overlap of genetic variation was found between ABB and IMB. Most of the remaining breeds also had their smallest *F*_*ST*_-value when compared to IMB. Only in BBV and DFV the smallest *F*_*ST*_-value was found compared to the alpine breeds OBV and FGV, respectively.

**Table 2:** **Pairwise *F***_***ST***_**-values estimated on 47,651 genotypes**

	**GLW**	**BBB**	**RH**	**BBV**	**OBV**	**MWF**	**FGV**	**DFV**	**ABB**	**IMB**
GLW	-	0.1274	0.1244	0.1576	0.1301	0.1345	0.1217	0.1313	0.1317	0.0959
BBB	0.1274	-	0.0792	0.1316	0.1040	0.1086	0.0931	0.1062	0.1009	0.0708
RH	0.1244	0.0792	-	0.1264	0.0997	0.1046	0.0914	0.1026	0.0938	0.0673
BBV	0.1576	0.1316	0.1264	-	0.0807	0.1058	0.0994	0.1026	0.1128	0.0852
OBV	0.1301	0.1040	0.0997	0.0807	-	0.0761	0.0698	0.0722	0.0845	0.0554
MWF	0.1345	0.1086	0.1046	0.1058	0.0761	-	0.0749	0.0779	0.0915	0.0617
FGV	0.1217	0.0931	0.0914	0.0994	0.0698	0.0749	-	0.0530	0.0819	0.0522
DFV	0.1313	0.1062	0.1026	0.1026	0.0722	0.0779	0.0530	-	0.0894	0.0604
ABB	0.1317	0.1009	0.0938	0.1128	0.0845	0.0915	0.0819	0.0894	-	0.0369
IMB	0.0959	0.0708	0.0673	0.0852	0.0554	0.0617	0.0522	0.0604	0.0369	-
Mean	0.1283	0.1024	0.0988	0.1113	0.0858	0.0928	0.0819	0.0884	0.0915	0.0651

### Confirmation of specific phenotypes

**Figure 2:**
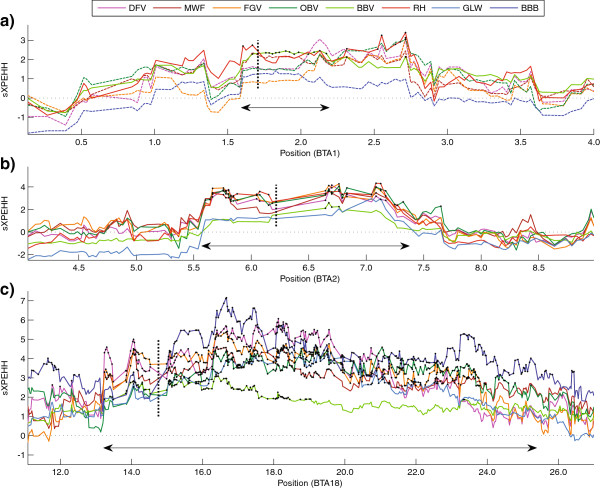
**Selection signatures around loci responsible for three distinct phenotypes used for confirmation.** Plot of sXPEHH values (y-axis) around the loci (x-axis in Mb) responsible for **a)** polledness in GLW **b)** double muscling in BBB and **c)** red coat colour in RH. A vertical dashed line marks the position of the target regions. Black asterisks mark significant SNPs. XP-EHH comparisons with control breeds that did not reach significance within a signature are in dashed lines. A double arrow marks the extensions of the detected signatures.

To assess the informative value of XP-EHH results we first checked if it was able to identify the selection signatures of three distinct phenotypes characteristic for one breed. These were polledness in GLW, double muscling in BBB and red coat colour in RH. The phenotypes double muscling and red coat colour are caused by variations in the genes *MSTN* on BTA2 [44] and *MC1R* on BTA18 [45], respectively. Though the underlying gene of the phenotype polledness in GLW is still unknown, we were recently able to identify a mutation on BTA1 in perfect association to it [46]. As Figure 2 shows, XP-EHH detected a significant selection signature at the respective genomic regions in each of the three breeds.

### Selection signatures close to known QTL

After conformation process of our XP-EHH-method that confirmed a selection signature for all three phenotypes tested, we checked if it was able to detect significant selection signatures close to previously published candidate genes of six known QTL associated with beef (*TG*) and dairy cattle production traits (*ABCG2*, Casein Cluster, *DGAT1*, *GH1*, *GHR*). As shown in Table 3, we found all six genes to be a part of selection signatures in at least one investigated cattle breed.

**Table 3:** Selection signatures around genes with known effect on milk and beef traits

**Gene**	**BTA**	**Position of target gene**	**Breed**	**Position of signature**	**SNP**	**Gene**
ABCG2	6	37,959,536-38,030,586	FGV	32,267,848-42,353,432	199	26
			MWF	36,697,298-42,548,023	146	22
			OBV	37,642,516-38,052,662	10	7
			BBV	37,897,068-38,052,662	3	3
Casein cluster	6	87,141,556-87,392,750	BBV	84,205,130-96,165,559	227	68
DGAT1	14	1,795,425-1,804,838	OBV	1-2,228,124	16	37
			MWF	1,524,578-2,205,696	12	34
TG	14	9,262,250-9,508,938	BBB	9,355,364-9,415,628	2	2
GH1	19	48,768,618-48,772,014	BBV	47,693,674-51,182,163	70	41
GHR	20	31,890,736-32,199,996	RH	28,500,780-39,168,038	195	50

Six target genes (Gene), their location on *Bos taurus* autosome (BTA) with physical position (bp) of the gene, the breed in which a selection signature was detected (Breed), physical position (bp) of the signature and number of SNPs (SNP) and genes (Gene) within the signature are given.

### Genome-wide selection signatures

In the artificially selected breeds we detected between 32 (OBV-MWF, GLW-MWF) and 217 (MWF-BBV) comparison-specific signatures including singletons, finally totalling and complementing to between 100 (OBV) and 229 (MWF) breed-specific confirmed signatures without singletons. The numbers of comparison-specific and breed-specific signatures as well as the average number of SNPs per signature are listed in Table 4.

**Table 4:** Selection signatures detected by XP-EHH for all ten breeds

**Control Breed**	**Case Breed**									
	**GLW**	**BBB**	**RH**	**BBV**	**OBV**	**MWF**	**FGV**	**DFV**	**ABB**	**IMB**
GLW	-	51	62	77	60	69	45	53	26	11
BBB	71	-	50	37	46	45	42	50	18	22
RH	63	76	-	82	44	90	58	70	16	33
BBV	139	117	130	-	62	217	118	115	23	14
OBV	86	78	64	118	-	76	63	82	25	19
MWF	32	60	42	39	32	-	47	64	18	18
FGV	65	66	52	95	49	75	-	73	20	19
DFV	65	80	72	100	45	79	67	-	19	18
Total ^a)^	199	232	217	201	175	414	290	269	123	129
Final	128	144	131	140	100	229	153	151	43	32
SNP/Signature	11.64	7.38	10.00	14.11	8.79	5.48	5.37	7.19	3.02	2.59
*N*_*e5*_	110.74	68.36	111.48	84.45	92.31	52.64	109.95	126.53	183.70	149.76
*N*_*e50*_	282.55	322.29	388.91	254.77	425.88	283.17	463.48	548.95	1680.69	1054.34
*N*_*e5*_/*N*_*e50*_	0.39	0.21	0.29	0.33	0.22	0.19	0.24	0.23	0.11	0.14

For each analysed breed (Case Breed) there is a column showing the number of significant selection signatures detected in comparison to the respective “Control Breed”, the number of significant selection signatures including singletons (Total), the number of signatures confirmed by neighbouring markers and/or multiple significant breed-comparisons (Final), the average number of SNPs per final signature (SNPs/Signature), the effective population sizes of 5 (*Ne*_*5*_), and 50 (*Ne*_*50*_) generations ago as well as their ratio (*Ne*_*5*_/*Ne*_*50*_).

^a)^As the same signature can be detected by comparison of one breed to several others, this is not simply the sum of all significant signatures.

To better understand the relationship between achieved XP-EHH results and genetic drift we estimated *N*_*e*_ of all ten breeds for 5 (*N*_*e5*_) to 50 (*N*_*e50*_) generations ago (Table 4) and tested their correlation with the number of detected signatures and the average number of SNPs per signature (Table 5). Generally, *N*_*e50*_, representing *N*_*e*_ at the time of the breed formation period (250 years ago assuming a 5-year generation interval in cattle), shows lower negative correlation to the number of detected confirmed signatures (-0.76) than the more recent *N*_*e*_ represented by *N*_*e5*_ (-0.82). The number of SNPs per signature shows stronger correlation with *N*_*e50*_ (-0.69) than with *N*_*e5*_ (-0.47). Interestingly, we found the strongest correlation (0.89) between the number of SNPs per signature and the recent decline in *N*_*e*_, represented by the *N*_*e5*_/*N*_*e50*_ ratio. In contrast, *N*_*e5*_/*N*_*e50*_ does not significantly affect the number of detected signatures in each breed (Table 5). The *N*_*e5*_/*N*_*e50*_ ratio equals 1.0 at constant *N*_*e*_ and approaches 0.0 with progressive reduction of *N*_*e*_ in the 45 considered generations. It is clearly evident from Table 4 that this ratio is small in the effectively still large artificially unselected IMB and ABB, which were around seven and nine times larger 45 generation ago. This corresponds to the progressive reduction of census population sizes in both non-commercial breeds during that time. On the other hand the *N*_*e5*_/*N*_*e50*_ ratio is relatively high in single purpose breeds GLW, BBV and RH, reflecting their relatively small *N*_*e*_ at breed formation period but also 5 generations ago. Therefore, high positive correlation (0.89) between signature lengths and the *N*_*e5*_/*N*_*e50*_ ratio points indirectly to the fact that highly selected (GLW, BBB, RH, BBV) and effectively smaller ($\bar{N_{e5}}$ = 93.7) breeds contain on average longer signatures (10.8 SNPs) than the two effectively larger ($\bar{N_{e5}}$ = 166.7) and artificially unselected breeds (IMB, ABB; 2.8 SNPs).

**Table 5:** Correlations between the effective population sizes and the number of detected signatures

	***N***_***e50***_	***N***_***e5***_	***N***_***e5***_ ***/N***_***e50***_
Total signatures	-0.57 (0.083)	-0.69 (0.028)	0.10 (0.788)
Final signatures	-0.76 (0.010)	-0.82 (0.004)	0.37 (0.290)
SNPs/Signature	-0.69 (0.026)	-0.47 (0.173)	0.89 (0.0004)

The correlation values are given along with the corresponding two-sided *P*-values in brackets. The correlations were estimated between the effective population sizes of 5 (*N*_*e5*_) and 50 (*N*_*e50*_) generations ago as well as the ratio of them (*N*_*e5*_/*N*_*e50*_) and the number of signatures including singletons (Total signatures), the number of confirmed signatures without singletons (Final signatures) and the average number of SNPs per final signature.

We investigated each confirmed selection signature by annotating the positional candidate genes. The (Additional file 4: Table S2, Additional file 5: Table S3, Additional file 6: Table S4, Additional file 7: Table S5, Additional file 8: Table S6, Additional file 9: Table S7, Additional file 10: Table S8, Additional file 11: Table S9, Additional file 12: Table S10 and Additional file 13: Table S11) show all breed-specific signatures with all positional candidate genes. As a small excerpt, some positional candidate genes of the most extended signature of each breed are listed in Table 6 and presented in the following results section in more detail. In addition, we show some striking, less extended selection signatures, each harbouring at most three positional candidate genes.

**Table 6:** Most extended selection signatures

**BTA**	**Breed**	**Start**	**End**	**SNP**	**Genes**	**Candidate genes mentioned in the text**
4	BBB	68,372,478	72,954,413	71	26	NPY
6	FGV	32,267,848	42,353,432	199	26	ABCG2, MEPE, IBSP, LAP3, NCAPG, LCORL
6	MWF	36,697,298	42,548,023	146	22	ABCG2, MEPE, IBSP, LAP3, NCAPG, LCORL
6	DFV	68,581,139	72,640,834	119	23	PDGFRA, KIT, KDR
6	BBV	84,205,130	96,165,559	227	68	BTC, ANKRD17, CSN1S1, CSN2, CSN1S2, CSN3, IL8
9	GLW	40,644,068	58,813,449	314	47	SIM1
11	OBV	64,291,486	71,493,179	130	38	PROKR1, GFPT1, GMCL1, PCBP1, EHD3
17	IMB	56,144,286	56,591,720	7	8	CAMKK2, ATP2A2
18	RH	13,218,147	25,386,867	231	85	MC1R, NKD1, NOD2, SALL1
20	ABB	2,571,860	3,171,036	9	4	NPM1, FGF18

The most extended signature of each of the ten breeds (Breed) is shown. *Bos taurus* autosome (BTA), the position in bp (Start to End) and the number of SNPs (SNP) per signature are given. From all positional candidate genes within the respective signature (Genes) only genes mentioned in the text are listed.

### Most extended selection signatures

For each of the ten breeds the most extended selection signature that recently underwent or is still undergoing strong positive selection was identified (Table 6). These ten signatures were distributed over seven chromosomes. In some instances the list of candidate genes is long and several of them are already associated with functional traits. In this case we only report the most plausible associations in the following lines. This approach of course does not exclude the possibility of not mentioned candidate genes being the true target of selection. The aim of the accompanied literature search [47] was primarily to analyse whether the detected selection signatures harbour some candidate genes that are already known to be associated with selected traits in cattle or other mammals.

The 600 kb region on BTA20, positively selected in ABB harbours four genes. Two of them have already been associated with traits: *NPM1* is potentially involved in diverse growth traits in Nanyang cattle at different ages [48,49] and *FGF18* is thought to influence atresia of follicles [50].

The most extended signature in IMB within 450 kb on BTA17 includes eight genes. Most outstanding are *CAMKK2* and *ATP2A2. CAMKK2* is thought to play a role in the luteolytic sensitivity of the bovine corpus luteum for PGF2alpha [51] whereas *ATP2A2* (alias *SERCA2*) seems to be involved in the regulation of calcium, particularly in the non-lactating mammary gland [52].

The most outstanding signature detected among all breeds spans about 18.2 Mb of BTA9 and is positively selected in GLW. Even though there is more than one promising candidate gene in this region, *single-minded (Drosophila) homologue 1* (*SIM1*) is a reasonable target for selection in a beef breed. *SIM1* is thought to regulate body weight and is associated with obesity in humans and mice (e.g. [53,54]) and was, therefore, declared to be one of the candidate genes for meat and carcass quality in cattle [55].

The second beef breed, BBB, shows a signature of positive selection on BTA4 in an interval covering approximately 4.6 Mb. The most striking positional candidate gene was *NPY*, which has already been associated with diverse growth traits in Nanyang cattle at different ages [56].

The positive selection signature on BTA6 in the dairy breed BBV (~12 Mb) encompasses 68 genes. Among these is *betacellulin* (*BTC*), shown to be expressed in a wide range of tissues, including the mammary gland. Moreover, it is suggested that the presence of *BTC* in milk might play a fundamental role in growth and development of neonates’ gastrointestinal tracts [57]. Another positional candidate gene, *ANKRD17*, has already been reported to be necessary for vascular maturation during embryonic development in mice [58]. Some additional quite auspicious genes were *IL8*, whose mutations were significantly associated with a substantial number of tested dairy traits in Chinese Holstein [59] and the genes of the casein-cluster *CSN1S1*, *CSN2*, *CSN1S2* and *CSN3*, which are associated with parameters like improved milk-clotting as well as protein and milk yield [60,61].

The selection signature on BTA18 in RH extends almost 12.2 Mb. Among the positional candidate genes there are four striking examples. Two can be roughly summarised as fertility related, *SALL1*[62] and *NKD1*[63], one (*NOD2* also named *CARD15*) is involved in dairy traits [64] and one (*MC1R* also *MSHR*) responsible for red coat colour [45].

The most extended selection signature of DFV was found on BTA6 spanning almost 4.1 Mb. The most outstanding genes of these regions were *PDGFRA*, *KIT* and *KDR*, which were declared to be candidate genes of selection signatures detected in different studies [10,15,20].

Interestingly FGV and MWF overlap in their most extended signatures on a region of almost 5.7 Mb. The other dual purpose breeds of alpine origin, DFV and OBV, as well as the closely related BBV also show selection signatures on the same part of BTA6, overlapping with the common section of FGV and MWF in almost 1.6 Mb. Therefore, the seven genes (*IBSP*, *MEPE*, *LAP3, MED28*, *DCAF16*, *NCAPG*, *LCORL*) lying in this area are assumed to be of special interest. Many of these candidate genes have already been associated with interesting traits. *IBSP* and *MEPE* were identified as possible candidates for protein and fat percentage in Israeli Holstein [65], but have also been associated with bone formation in humans [66] and declared as candidate genes for dystocia and stillbirth in Norwegian Red cattle [67]. SNPs in *LAP3* have been shown to impact fat as well as protein percentage and milk somatic cell score [68]. *NCAPG* was associated with bovine carcass weight [69], fetal growth [70] as well as increased body frame size at puberty in cattle [71]. Moreover, Lindholm-Perry *et al.*[72] found a significant association of several makers within the *NCPAG-LCORL* locus with feed intake and body weight traits.

Of the 38 genes covered by the most extended signature in OBV, that is spanning 7.2 Mb on BTA11, five genes have already been associated with fertility traits: *PROKR1*[73], *GFPT1*[74], *EHD3*[75], *GMCL1*[76] and *PCBP1*[77,78].

The above highlights most extended signatures of all ten breeds likely to represent the strongest selected chromosomal regions and harbour the genetic background of strongly desired phenotypes. Due to, in particularly, the enormous extensions of these signatures, identifying their causal gene(s) will be challenging. More promising for the deciphering of new adaptive pathways will be the investigation of less extended signatures that incorporate only a few or even just one positional candidate gene like those presented in the subsequent section.

### Some striking but less extended selection signatures

We investigated less extended signatures harbouring only one to three positional candidate genes (named “short signatures” in the following) for possible associations cited in literature. In doing so, we were able to identify about 80 potential candidate genes that have been associated or suspected to be related to important traits in human, goat, pig, cattle and other species (Additional file 14: Table S12). Most of them can be classified in a broader sense to one of the following traits: fertility, immunology, personality, pigmentation, milk, and the lion’s share belonging to body weight, carcass and growth. Predominantly, these candidate genes were part of short signatures in one single breed. Only eleven candidate genes could be detected in short signatures in two breeds.

**Figure 3:**
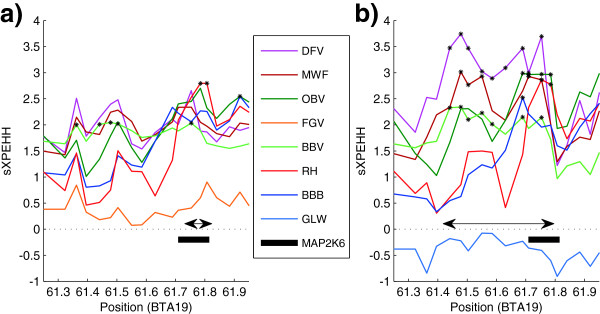
**Selection signature around *MAP2K6 *in GLW and FGV.** Plot of the sXPEHH values (y-axis) of two detected selection signatures around *MAP2K6* (x-axis in Mb) in **a)** GLW and **b)** FGV. Asterisks mark significant SNPs. A double arrow marks the extensions of the detected signatures.

One of those short signatures includes *MAP2K6* (associated with carcass weight, back fat thickness and marbling score in Korean beef cattle [79]) on BTA19 and was found in GLW and FGV. The selection signature around *MAP2K6* is shown in Figure 3 for GLW and FGV. In both breeds there is no further gene annotated in the respective signature. In GLW the signature is almost completely restricted to the position of *MAP2K6* whereas it is more extended in FGV. However, the curve progression of the sXPEHH values in FGV seems to be bimodal with one peak at the end of the signature (position of *MAP2K6*) and the other at the beginning. At this location there is also a selection signature in GLW but, unlike to FGV, it is a discrete signature that is separated from the one around *MAP2K6*. This shows possible evidence for the signature in FGV to be due to two different targets of selection.

All selection signatures and positional candidate genes are listed in the breed specific additional files (Additional file 4: Table S2, Additional file 5: Table S3, Additional file 6: Table S4, Additional file 7: Table S5, Additional file 8: Table S6, Additional file 9: Table S7, Additional file 10: Table S8, Additional file 11: Table S9, Additional file 12: Table S10 and Additional file 13: Table S11). These tables give detailed information on the genomic position, extension, breed-comparison that detected the signature and all additional positional candidate genes within each signature.

## Discussion

The adaptation to new environments and artificial selection based on distinct breeding goals has shaped the phenotypic appearance of cattle since domestication. The Industrial Revolution with growing city populations stimulated the development of specialised dairy, beef and dual-purpose breeds at turn of the 18^th^ to 19^th^ century [25]. This progressive breed differentiation was accelerated by introduction of breed standards, which accumulated specific phenotypes that visibly distinguished particular breeds. Parallel introduction of herdbooks reduced gene flow between breeds. The recent advances in quantitative genetics and reproductive techniques have enabled the application of enormous selection intensity within particular cattle breeds. Uncovering the genetic footprints of this strong recent artificial selection could give an insight into the mechanisms of selection in general and could, moreover, help to assign chromosomal regions related to important physiological and economical traits. With this aim, we performed a genome-wide scan of recent artificial selection in eight selected and two mainly artificially unselected cattle breeds using XP-EHH.

To avoid population stratifications within the sets of animals and to be able to assess the genetic diversity of the ten breeds, we performed PCA and pair-wise *F*_*ST*_-estimations before conducting XP-EHH analyses. Taken together, the PCA- and *F*_*ST*_-results imply that the two artificially unselected breeds are not well differentiated and still cover a considerable part of the original genetic diversity. In contrast, artificially selected breeds show significantly higher differentiation, e.g. the average *F*_*ST*_-value for GLW (0.128) is around twice the average for IMB (0.065, Table 2). These results confirm findings of our previous diversity studies based on microsatellite markers [27,28].

Using XP-EHH we were able to identify between 32 (IMB) and 229 (MWF) selection signatures per breed and some promising candidate genes. The overlap with other studies was only moderate. This moderate agreement was no surprise at all and might be due to a number of parameters. One is the discrepancy in the power of methods used to identify selection signatures in different designs. Since this is the first genome-wide study using XP-EHH in cattle it is not possible to compare the results to another study utilizing the same method. Some previous studies based on long haplotypes using methods like EHH [19] or iHS [13] were looking for selection within and not between breeds. The applied methods in these studies have low power or are even unable to detect signatures of selected alleles at high frequency (>0.8) [26]. Moreover, the breeds investigated as well as the design of investigation (breeds separately vs. breeds with similar breeding purpose combined to one group) have major impact on the results. However, if we compare our results to that of Gautier and Naves [20], who detected 23 selection signatures in Creole Cattle using average ancestry methods, iHS and especially Rsb, which is similarly to XP-EHH a haplotype based method for across population study [18], we found considerable overlap. They detected 19 of the 23 signatures using the Rsb approach and were able to define candidate genes for 11 [20]. About one third (*PDE1B*, *KDR*, *SAMD12* and *SLC7A5*) were also declared as positional candidate genes in at least one of the investigated breeds in our study. The Creole cattle represents an admixture of European, African and zebu cattle kept in tropical environments. However, pure European cattle breeds investigated in this case are mainly kept in -and are therefore adapted to- a temperate climate. Considering these discrepancies, the overlap in question is quite remarkable. Moreover, corresponding signatures can be assumed to represent more adaptions to selective pressures concerning performance than environment.

Additional reasons for divergences from previous studies or our own expectations may be due to the discrepancies in SNP densities and animal numbers that might influence the accuracy of results. For example, the surrounding SNPs of *MSTN* and *MC1R* (two obvious targets of selection) were more than 520 kb (*MSTN*) respectively 490 kb (*MC1R*) apart. Such huge gaps can bias haplotyping and the XP-EHH estimation on both sides of the gap thus precluding an adequate signature mapping within the gap. Although both, *MSTN* and *MC1R*, are surrounded by significant SNPs it is obvious (Figure 2) that they seem to lie in a sXPEHH depression compared to a little more distant SNPs. Therefore, especially for less distinctive signatures a similar SNP distribution might prevent its detection or at least disjoint an otherwise consistent signature.

In general, a high level of neutral genetic diversity was assumed in the effectively large ancestral population of the wild progenitors [23]. Domestication eventually caused some variants to become quite advantageous in a possibly very small founder population [80]. Even if these variants had only reached the frequency of 0.01 in the effectively very large wild auerochs population the “derived” allele could have resided within different haplotypes. If more than one of those haplotypes passed the domestication bottleneck and survived until the period of breed formation it would no longer match the “classical selective sweep” model. This circumstance complicates their detection with diverse methods, especially if the initial allele frequency was already moderate [23]. Our design is optimised to detect the more recently selected variants involved in breed specific phenotypes and improved performance of divergently selected breeds. Although genomic scans in our design can yield an unrepresentative subset of loci that contribute to genetic gains or adaptations [81] we do not expect an increase of false positives due to predominant recent positive selection on standing variation [23,81].

The intensity of the artificial selection is inevitably associated with the number of parents of the next generation [82]. This is reflected in both, current effective population size (e.g. *N*_*e5*_) and number of positively selected sites (Table 4). Therefore, negative correlation between *N*_*e5*_ and the number as well as the extent of selection signatures is expected and cannot be considered as evidence for mapping of genomic regions due to drift alone. Admittedly, with further reduction in sample size the estimate of *N*_*e*_ gets simultaneously smaller. This means that the varying sample sizes (31 for BBB up to 50 for DFV, FGV, BBV and RH) are potentially able to affect *N*_*e*_-results. However, sample sizes are not chosen freely but dependent on the degree of familial relationship within the sampled subpopulations as measured by UAR. Filtering subpopulations using UAR will reduce the sample of populations that have a smaller *N*_*e*_ more than those with higher *N*_*e*_, since individuals in effectively small populations are in general closer related. The observed relationship between estimated *N*_*e*_ and sample size might thus be a result of UAR filtering that determined sample size. Taken together, we conclude that the estimated *N*_*e*_ is related to the true *N*_*e*_. This conclusion is strongly supported by the fact that the estimated *N*_*e*_–values match with our expectations especially regarding the relative sizes and recent demographical processes of the investigated breeds. Therefore, we assumed that different sample size or *N*_*e*_ do not bias our design although great differences between compared breeds might not be advantageous. However, the final number of selection signatures after excluding all singletons was more strongly correlated with estimated *N*_*e*_ and the estimated negative correlation was even stronger for *N*_*e5*_ than *N*_*e50*_. Since *N*_*e50*_ coincides with the breed founding period, here detected selection signatures reflect a combination of breed specific phenotypes and quantitative traits under partly divergent artificial selection in artificially isolated subpopulations.

Regardless of whether breed characteristics or quantitative traits are predominantly mapped, the main question is where to set the significance threshold in order to get as few as possible false positives due to genetic drift, while retaining as much as possible of the truly selected sites. Unlike for human evolution, a precise demographic model as a basis for the coalescence simulation is not available for the diversification of cattle breeds. But even for human data there is considerable uncertainty in the models simulated, which makes the usefulness of assigning formal *p*-values debatable [17,36]. Therefore, many studies in both, human and animal data, set the significance threshold empirically (e.g. [12,13,17,19,20,36,83,84]). For this, the thresholds are predominantly set freely without any relation to real or simulated data (distinct percentage of most extreme values in the empirical distribution). This is the reason why alternative approaches for the determination of the significance threshold are needed. For this purpose, we introduced the usage of artificially unselected breeds for the determination of this threshold. It is quite clear to us that the proposed thresholds have no formal statistical validity but in comparison to arbitrary empirical thresholds we consider our solution as an improvement for domesticated species without living wild progenitor. We tested this approach by detecting the selection signatures around three distinct phenotypes typical for one individual breed (polledness in GLW, double muscling in BBB and red coat colour in RH). At first sight it might be surprising that the selection signature in GLW was only found in comparison with two breeds (RH, BBV) since all investigated breeds, excluding GLW, are horned. However, we demonstrated in two previous studies that the polled mutation resides on the most frequent haplotype over all breeds [46,85]. This reduced haplotype diversity around the polled locus might be due to an ancient selection signature prior to breed formation and prohibits a distinct signature mapping for at least some control breeds. Similar selective sweeps present in all investigated subpopulations might therefore be missed by XP-EHH. It thus affirms our approach that the polled signature in GLW could nonetheless be detected in two comparisons (RH and BBV). Although further improvements to our significance determination approach are possible, we believe that it is a promising step when it comes to keeping false-positive rates under control.

The approach of proposing candidate genes in the vicinity of the peak location within a selection signature represents a common way to identify a possible candidate gene (e.g. [10]). However, especially in quite extended signatures that incorporate a lot of genes and are therefore likely to occupy more than one target of positive selection, promising candidate genes might be lost. This is the main reason why we decided against this approach and reported all positional candidate genes (Additional file 4: Table S2, Additional file 5: Table S3, Additional file 6: Table S4, Additional file 7: Table S5, Additional file 8: Table S6, Additional file 9: Table S7, Additional file 10: Table S8, Additional file 11: Table S9, Additional file 12: Table S10 and Additional file 13: Table S11). The circumstance of a possible bimodal signature had already been mentioned when presenting the selection signature around *MAP2K6* (Figure 3).

We also detected a number of signatures (480 over all ten breeds) that did not incorporate any gene at all. This is not new in genome-wide searches for signatures of selection and has been discussed in human studies [4] as well as in livestock animals [10,83,84,86]. In some cases the selected gene might be just a few kb outside the determined signature, some might be false positives but for most of these regions the underlying selected structures might simply not be annotated or even not known as yet. There are highly significant and by multiple comparisons confirmed signatures without any known gene inside. For example, we detected a signature in the case breed GLW (BTA7: 79,494,846-80,987,053) only but it was confirmed five times (significant in comparison to five “control breeds”). Additionally, there are signatures detected at the same position in multiple case breeds (e.g. a common significant region for BBB, DFV and IMB on BTA9: 12,134,014-12,252,182). Such analogies within and between case breeds are unlikely to occur only by chance due to random drift. Instead, it is more likely these signatures harbour yet unknown genes. It is certainly intriguing that we found 97 of the 480 signatures without annotated genes to be overlapping between at least two breeds in 45 distinct regions.

In general, combining different methods of detecting selection signatures [7] or combining the results of selection signature and association studies [87] might improve resolution of quite extended signatures and reduce the number of false positives. However, the information gained by such combining methods will always depend on the existence and use of the correct phenotype shaped by the selective pressure. Setting up correct adaptation hypotheses for signatures without genes or signatures based on traits not yet phenotyped, will keep scientists engaged in the coming decade. Single broad selection signatures could include closely linked genes affecting, for instance, important yield traits, coat colour and resistances all differentially selected in the compared breeds. Successive association studies considering the appropriate traits in appropriate models could be able to decipher such a complex and broad selection signature signal into involved phenotypes.

Many differentially selected phenotypes within cattle breeds were the subject of selection for novelty. This practice was common due to the human tendency to select phenotypes visibly distinguishing particular breeds. We adapted a design so that it should be able to detect a large fraction of differentially selected breed characteristics as well as differentially selected (quantitative) traits. Nevertheless, a final validation for both the way of defining the significance thresholds based on artificially unselected breeds as well as for the found signatures of selection will only be possible when further studies are performed and some candidate genes are irrevocably identified. This proof of causality will be easier to achieve for less extended signatures that include only a few or just one candidate gene. The experiences gathered by resolving these “simpler” signatures might in turn lay the foundation for dissecting the more extended signatures.

## Conclusions

Our results demonstrate that the XP-EHH approach can be very useful for mapping selection signatures in artificially selected breeds of livestock animals. Specific genetic architecture could make the detection of selection signatures in domesticated animals (artificially isolated and intensively selected) more rewarding and less costly than in humans even where classic sweeps were not a dominant mode of adaptation after domestication 10,000 years ago. The determination of the significance threshold in studies of selection signatures within and between breeds of domesticated species is still a major challenge. We made use of the artificially unselected breeds ABB and IMB to account for the maximum XP-EHH value that seems to be achievable if genetic drift and natural or weak artificial selection are acting only. In doing so we were able to detect up to 229 selection signatures per breed, thereby recovering a number of genes among the selected sites that are already known to be involved in important breeding traits. Nevertheless, distinguishing the effects of positive selection from those of population demographics or genetic drift is still a major challenge that needs to be resolved. We found hundreds of signatures without any genes. About one fifth of those were mapped in more than one breed and even more (~40%) reached significance in the respective case breed with more than one control breed. The proof of causality for most significant signatures with obvious candidate genes and the setting up of a correct adaptation hypothesis for signatures without genes present further challenges in the effort to uncover new adaptive pathways. Our results clearly highlight numerous genes associated with important traits and characteristics selected within breeds or breed groups. These might eventually contribute to the identification of the selected variants that caused the signatures found. In most cases advanced studies will be needed to distinguish between signatures caused by breed specific characteristics or traits of practical interest for agriculture. Both are of interest for a better understanding of mechanisms and targets of artificial selection in species of domesticated animals.

## Abbreviations

EHH: Extended haplotype homozygosity; iHS: Integrated haplotype score; XP-EHH: Cross population extended haplotype homozygosity; sXPEHH: Standardised XP-EHH values; Rsb: Relative integrated EHH of a site between populations; FST: Population fixation index; ABB: Anatolian Black; IMB: Illyrian Mountain Buša; BBB: Blanc-Bleu Belge; GLW: Galloway; RH: Red Holstein; BBV: Braunvieh; OBV: Original Braunvieh; MWF: Murnau-Werdenfelser; DFV: German Fleckvieh; FGV: Franken Gelbvieh; Na: Number of animals of a breed used for selection signature analyses; Ng: Number of sampled individuals of a breed; UAR: Unified additive relationship; maxABB: Chromosomal maximum sXPEHH value of the case breed ABB compared to a specific control breed; maxIMB: Chromosomal maximum sXPEHH value of the case breed IMB compared to a specific control breed, gene symbols are given according to the UCSC Genome Browser.

## Competing interests

The authors declare that they have no competing interests.

## Authors’ contributions

SR assisted in designing experiments, carried out principal component analysis, estimation of pairwise *F*_*ST*_ values, XP-EHH- and LD-analyses as well as *N*_*e*_–estimations, contributed to results interpretation and drafted the manuscript. IM designed experiments, supervised the study, organized sampling, performed plausibility control of the data, supplied database applications for all the subsequent data analyses, partly performed data analyses, contributed to the interpretation of data, revised and edited the manuscript. DS generated and performed the initial analysis of the genotyping data. MF supplied material and critically revised the manuscript. All authors read and approved the final manuscript.

## Supplementary Material

Additional file 1: Figure S1 Determination of significance thresholds. The PDF illustrates the determination of the significance threshold for DFV in comparison to RH on *Bos taurus* autosome (BTA) 6. A) The distribution of the sXPEHH values of BTA6 in the IMB-RH comparison. According to the convention used in the sXPEHH-program, the positive sXPEHH values suggest selective pressure in the first breed of comparison IMB-RH (i.e. IMB) while the negative values suggest selective pressure in the second breed (i.e. RH). The maximal positive sXPEHH values of the comparison IMB-RH (maxIMB: 2.658523) points to a SNP under possible selection pressure in artificially unselected population IMB. B) Similar to the above, the maximal positive sXPEHH values of comparison ABB-RH (maxABB: 3.056731) points to a SNP under possible selective pressure in the artificially unselected population ABB. C) The distribution of the sXPEHH values of BTA6 in the DFV-RH comparison. The chromosome-wide significance threshold for XP-EHH comparisons of the control breed RH with any other artificially selected breed, e.g. DFV, is defined as the mean value of maxIMB and maxABB (2.857627). All sXPEHH values above this threshold are declared significant for selection in DFV if contrasted to RH on BTA6 and shaded in red. Click here for file

Additional file 2: Table S1 Significance thresholds for all autosomes. This excel file shows the significance thresholds for all 29 *Bos taurus* autosomes (BTA) and all eight artificially selected breeds (breed abbreviations). Each breed represents the respective control breed for XP-EHH calculation. If a marker in a XP-EHH comparison reaches a sXPEHH value above the threshold of the control breed on the specific chromosome it is called significant. These thresholds were calculated as the mean of the chromosome-wide maximum values reached by ABB and IMB (maxABB; maxIMB) when calculating XP-EHH with the respective control breed (Additional file 1: Figure S1). Click here for file

Additional file 3: Figure S2 Extent-determining of selection signatures. The PDF illustrates the simulated sequence of 22 SNPs, where a red star marks significant SNPs and a blue dot non-significant. The rows A), B) and C) represent all assumed breed-comparisons of DFV for which at least one of the 22 SNPs was significant. Now each SNP of the comparison-independent row D) that represents DFV in total is significant if the respective SNP is significant in at least one breed-comparison A), B) or C). All remaining SNPs stay non-significant. All significant SNPs belong to the same selection signature if they are not separated by more than one non-significant SNP in D). Finally, the signature is extended at each side by half the distance to the neighbouring non-significant SNPs (marked with a green dashed line) to get the final signature of selection spanning the distance marked by a double arrow. Click here for file

Additional file 4: Table S2 Significant markers of the breed Galloway. Listed are all significant markers with their SNP-Number (SNP-No), representing the range of the respective SNPs within all 47,651 SNPs ordered per chromosome and position, the SNP position and corresponding *Bos taurus* autosom (BTA). The breed-abbreviations represent the respective control breed (DFV: German Fleckvieh, OBV: Original Braunvieh, MWF: Murnau-Werdenfelser, FGV: Franken Gelbvieh, RH: Red Holstein, BBV: Braunvieh, BBB: Blanc-Bleu Belge). In case of a significant comparison to the respective control breed the sXPEHH-value is shown in these columns, otherwise the field is left empty. *nSC* shows the overall number of significant breed comparisons for each marker. Furthermore, all genes within the signature, which starts at position (bp) “Start of Sig” and ends at “End of Sig” according to the UCSC Genome Browser, are listed. Click here for file

Additional file 5: Table S3 Significant markers of the breed Blanc-Bleu Belge. Listed are all significant markers with their SNP-Number (SNP-No), representing the range of the respective SNPs within all 47,651 SNPs ordered per chromosome and position, the SNP position and corresponding *Bos taurus* autosom (BTA). The breed-abbreviations represent the respective control breed (DFV: German Fleckvieh, OBV: Original Braunvieh, MWF: Murnau-Werdenfelser, FGV: Franken Gelbvieh, RH: Red Holstein, BBV: Braunvieh, GLW: Galloway). In case of a significant comparison to the respective control breed the sXPEHH-value is shown in these columns, otherwise the field is left empty. *nSC* shows the overall number of significant breed comparisons for each marker. Furthermore, all genes within the signature, which starts at position (bp) “Start of Sig” and ends at “End of Sig” according to the UCSC Genome Browser, are listed. Click here for file

Additional file 6: Table S4 Significant markers of the breed Red Holstein. Listed are all significant markers with their SNP-Number (SNP-No), representing the range of the respective SNPs within all 47,651 SNPs ordered per chromosome and position, the SNP position and corresponding *Bos taurus* autosom (BTA). The breed-abbreviations represent the respective control breed (DFV: German Fleckvieh, OBV: Original Braunvieh, MWF: Murnau-Werdenfelser, FGV: Franken Gelbvieh, BBV: Braunvieh, GLW: Galloway, BBB: Blanc-Bleu Belge). In case of a significant comparison to the respective control breed the sXPEHH-value is shown in these columns, otherwise the field is left empty. *nSC* shows the overall number of significant breed comparisons for each marker. Furthermore, all genes within the signature, which starts at position (bp) “Start of Sig” and ends at “End of Sig” according to the UCSC Genome Browser, are listed. Click here for file

Additional file 7: Table S5 Significant markers of the breed Braunvieh. Listed are all significant markers with their SNP-Number (SNP-No), representing the range of the respective SNPs within all 47,651 SNPs ordered per chromosome and position, the SNP position and corresponding *Bos taurus* autosom (BTA). The breed-abbreviations represent the respective control breed (DFV: German Fleckvieh, OBV: Original Braunvieh, MWF: Murnau-Werdenfelser, FGV: Franken Gelbvieh, RH: Red Holstein, GLW: Galloway, BBB: Blanc-Bleu Belge). In case of a significant comparison to the respective control breed the sXPEHH-value is shown in these columns, otherwise the field is left empty. *nSC* shows the overall number of significant breed comparisons for each marker. Furthermore, all genes within the signature, which starts at position (bp) “Start of Sig” and ends at “End of Sig” according to the UCSC Genome Browser, are listed. Click here for file

Additional file 8: Table S6 Significant markers of the breed Original Braunvieh. Listed are all significant markers with their SNP-Number (SNP-No), representing the range of the respective SNPs within all 47,651 SNPs ordered per chromosome and position, the SNP position and corresponding *Bos taurus* autosom (BTA). The breed-abbreviations represent the respective control breed (DFV: German Fleckvieh, MWF: Murnau-Werdenfelser, FGV: Franken Gelbvieh, RH: Red Holstein, BBV: Braunvieh, GLW: Galloway, BBB: Blanc-Bleu Belge). In case of a significant comparison to the respective control breed the sXPEHH-value is shown in these columns, otherwise the field is left empty. *nSC* shows the overall number of significant breed comparisons for each marker. Furthermore, all genes within the signature, which starts at position (bp) “Start of Sig” and ends at “End of Sig” according to the UCSC Genome Browser, are listed. Click here for file

Additional file 9: Table S7 Significant markers of the breed Murnau-Werdenfelser. Listed are all significant markers with their SNP-Number (SNP-No), representing the range of the respective SNPs within all 47,651 SNPs ordered per chromosome and position, the SNP position and corresponding *Bos taurus* autosom (BTA). The breed-abbreviations represent the respective control breed (DFV: German Fleckvieh, OBV: Original Braunvieh, FGV: Franken Gelbvieh, RH: Red Holstein, BBV: Braunvieh, GLW: Galloway, BBB: Blanc-Bleu Belge). In case of a significant comparison to the respective control breed the sXPEHH-value is shown in these columns, otherwise the field is left empty. *nSC* shows the overall number of significant breed comparisons for each marker. Furthermore, all genes within the signature, which starts at position (bp) “Start of Sig” and ends at “End of Sig” according to the UCSC Genome Browser, are listed. Click here for file

Additional file 10: Table S8 Significant markers of the breed Franken Gelbvieh. Listed are all significant markers with their SNP-Number (SNP-No), representing the range of the respective SNPs within all 47,651 SNPs ordered per chromosome and position, the SNP position and corresponding *Bos taurus* autosom (BTA). The breed-abbreviations represent the respective control breed (DFV: German Fleckvieh, OBV: Original Braunvieh, MWF: Murnau-Werdenfelser, RH: Red Holstein, BBV: Braunvieh, GLW: Galloway, BBB: Blanc-Bleu Belge). In case of a significant comparison to the respective control breed the sXPEHH-value is shown in these columns, otherwise the field is left empty. *nSC* shows the overall number of significant breed comparisons for each marker. Furthermore, all genes within the signature, which starts at position (bp) “Start of Sig” and ends at “End of Sig” according to the UCSC Genome Browser, are listed. Click here for file

Additional file 11: Table S9 Significant markers of the breed German Fleckvieh. Listed are all significant markers with their SNP-Number (SNP-No), representing the range of the respective SNPs within all 47,651 SNPs ordered per chromosome and position, the SNP position and corresponding *Bos taurus* autosom (BTA). The breed-abbreviations represent the respective control breed (OBV: Original Braunvieh, MWF: Murnau-Werdenfelser, FGV: Franken Gelbvieh, RH: Red Holstein, BBV: Braunvieh, GLW: Galloway, BBB: Blanc-Bleu Belge). In case of a significant comparison to the respective control breed the sXPEHH-value is shown in these columns, otherwise the field is left empty. *nSC* shows the overall number of significant breed comparisons for each marker. Furthermore, all genes within the signature, which starts at position (bp) “Start of Sig” and ends at “End of Sig” according to the UCSC Genome Browser, are listed. Click here for file

Additional file 12: Table S10 Significant markers of the breed Anatolian Black. Listed are all significant markers with their SNP-Number (SNP-No), representing the range of the respective SNPs within all 47,651 SNPs ordered per chromosome and position, the SNP position and corresponding *Bos taurus* autosom (BTA). The breed-abbreviations represent the respective control breed (DFV: German Fleckvieh, OBV: Original Braunvieh, MWF: Murnau-Werdenfelser, FGV: Franken Gelbvieh, RH: Red Holstein, BBV: Braunvieh, GLW: Galloway, BBB: Blanc-Bleu Belge). In case of a significant comparison to the respective control breed the sXPEHH-value is shown in these columns, otherwise the field is left empty. *nSC* shows the overall number of significant breed comparisons for each marker. Furthermore, all genes within the signature, which starts at position (bp) “Start of Sig” and ends at “End of Sig” according to the UCSC Genome Browser, are listed. Click here for file

Additional file 13: Table S11 Significant markers of the breed Illyrian Mountain Buša. Listed are all significant markers with their SNP-Number (SNP-No), representing the range of the respective SNPs within all 47,651 SNPs ordered per chromosome and position, the SNP position and corresponding *Bos taurus* autosom (BTA). The breed-abbreviations represent the respective control breed (DFV: German Fleckvieh, OBV: Original Braunvieh, MWF: Murnau-Werdenfelser, FGV: Franken Gelbvieh, RH: Red Holstein, BBV: Braunvieh, GLW: Galloway, BBB: Blanc-Bleu Belge). In case of a significant comparison to the respective control breed the sXPEHH-value is shown in these columns, otherwise the field is left empty. *nSC* shows the overall number of significant breed comparisons for each marker. Furthermore, all genes within the signature, which starts at position (bp) “Start of Sig” and ends at “End of Sig” according to the UCSC Genome Browser, are listed. Click here for file

Additional file 14: Table S12 Candidate genes of short signatures. This excel file shows candidate genes of short signatures (that means at most three annotated genes) found on a given chromosome (BTA) in the respective case breed. All listed genes have already been associated or at least suspected to be involved in interesting traits and metabolic pathways in different species. The respective trait/pathway as well as the species it was investigated in and the corresponding reference are given. Click here for file
