# Supplementary figures and images for: A genome-wide scan for signatures of differential artificial selection in ten cattle breeds
Source: BMC Genomics. 2013 Dec 21;14:908. doi: 10.1186/1471-2164-14-908 (PMC3878089; doi:10.1186/1471-2164-14-908)

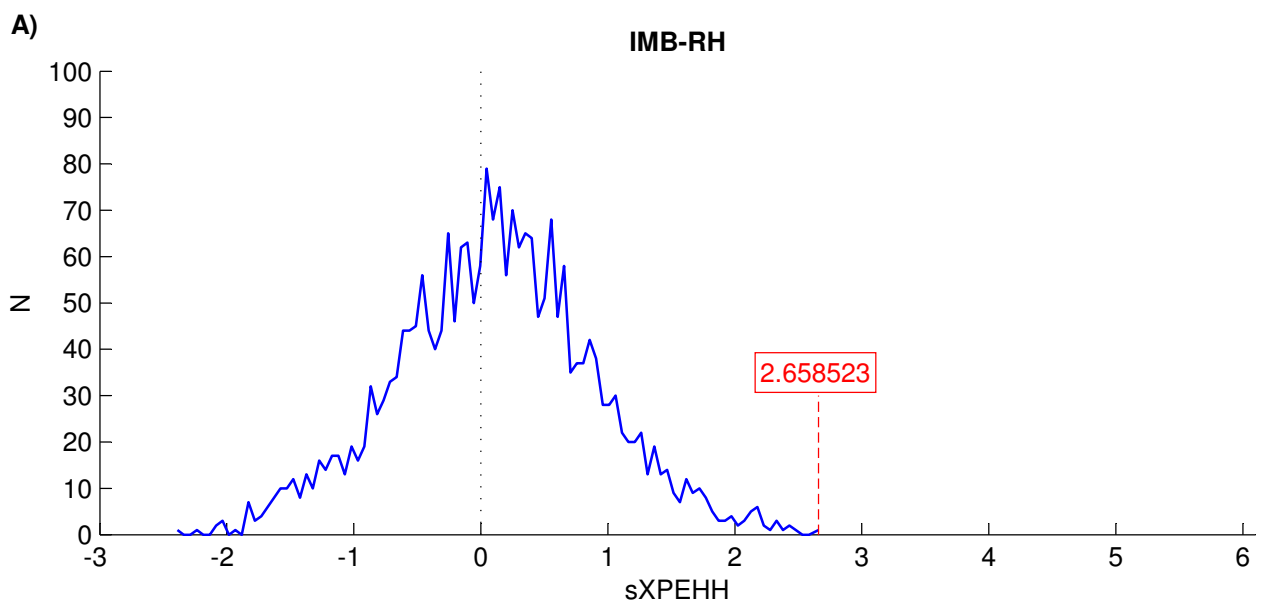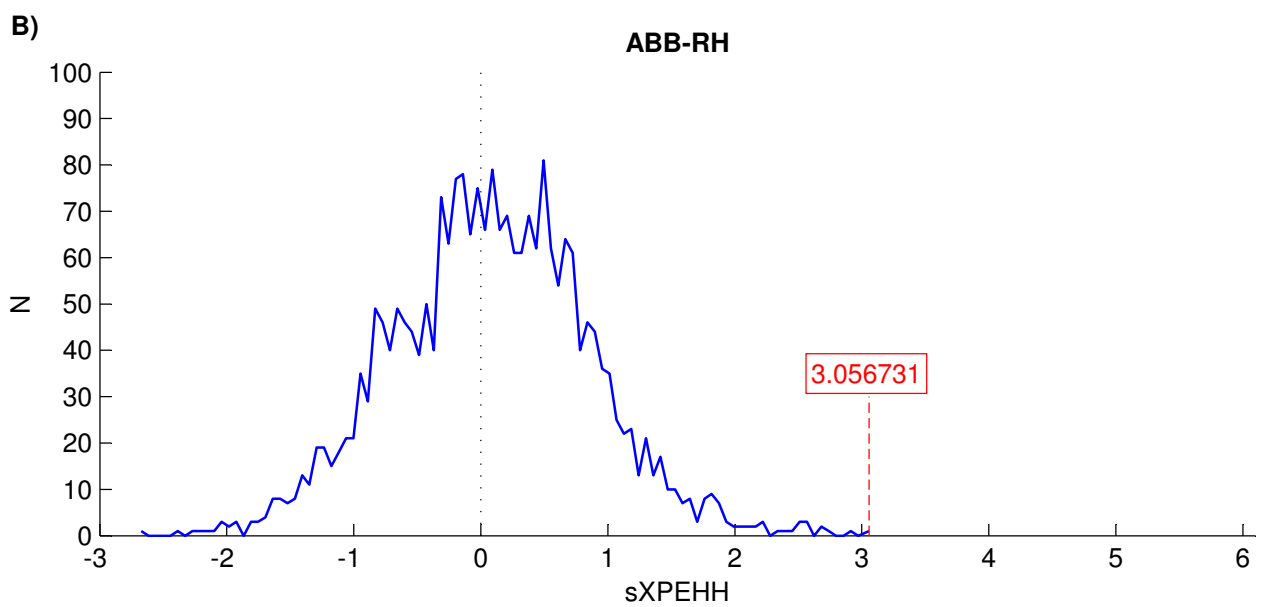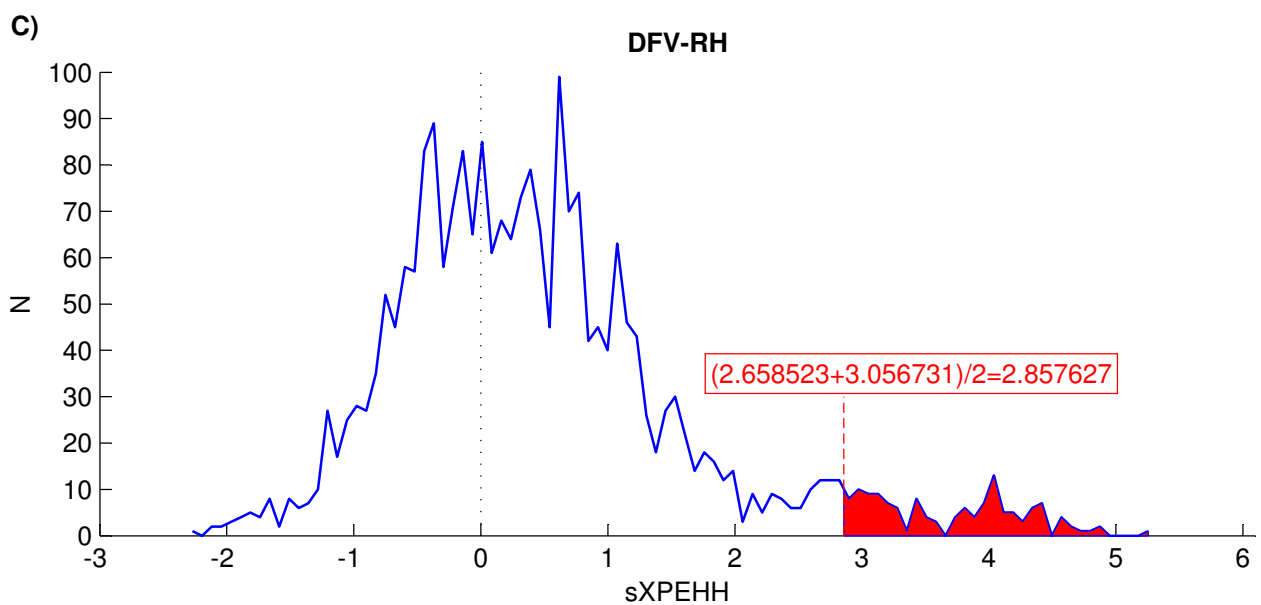

Supplement: Additional file 1: Figure S1 — Determination of significance thresholds. The PDF illustrates the determination of the significance threshold for DFV in comparison to RH on Bos taurus autosome (BTA) 6. A) The distribution of the sXPEHH values of BTA6 in the IMB-RH comparison. According to the convention used in the sXPEHH-program, the positive sXPEHH values suggest selective pressure in the first breed of comparison IMB-RH (i.e. IMB) while the negative values suggest selective pressure in the second breed (i.e. RH). The maximal positive sXPEHH values of the comparison IMB-RH (maxIMB: 2.658523) points to a SNP under possible selection pressure in artificially unselected population IMB. B) Similar to the above, the maximal positive sXPEHH values of comparison ABB-RH (maxABB: 3.056731) points to a SNP under possible selective pressure in the artificially unselected population ABB. C) The distribution of the sXPEHH values of BTA6 in the DFV-RH comparison. The chromosome-wide significance threshold for XP-EHH comparisons of the control breed RH with any other artificially selected breed, e.g. DFV, is defined as the mean value of maxIMB and maxABB (2.857627). All sXPEHH values above this threshold are declared significant for selection in DFV if contrasted to RH on BTA6 and shaded in red. [file 1471-2164-14-908-S1.pdf]

A) DFV-BBB

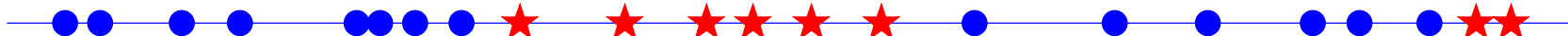

B) DFV-OBV

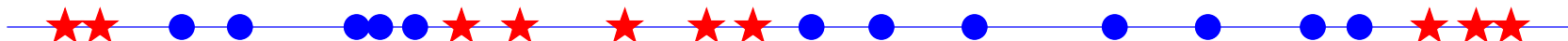

C) DFV-RH

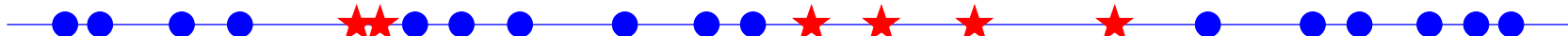

D) DFV

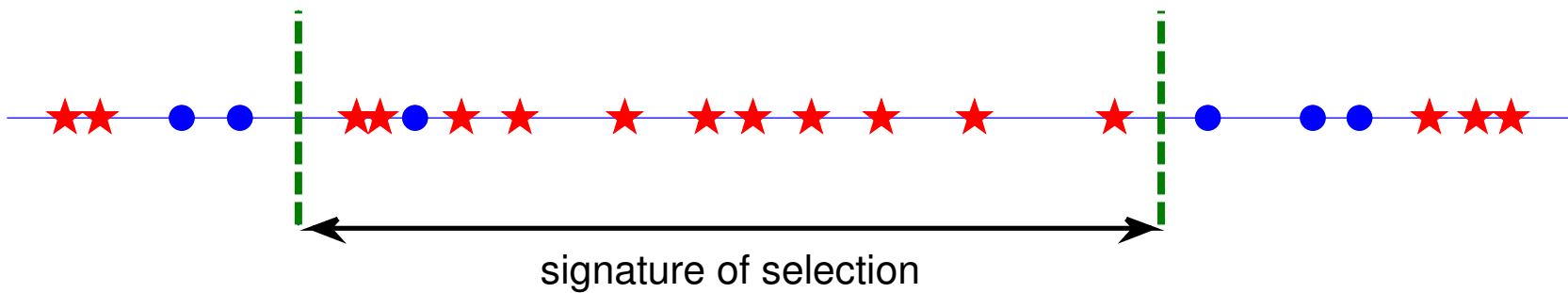

Supplement: Additional file 3: Figure S2 — Extent-determining of selection signatures. The PDF illustrates the simulated sequence of 22 SNPs, where a red star marks significant SNPs and a blue dot non-significant. The rows A), B) and C) represent all assumed breed-comparisons of DFV for which at least one of the 22 SNPs was significant. Now each SNP of the comparison-independent row D) that represents DFV in total is significant if the respective SNP is significant in at least one breed-comparison A), B) or C). All remaining SNPs stay non-significant. All significant SNPs belong to the same selection signature if they are not separated by more than one non-significant SNP in D). Finally, the signature is extended at each side by half the distance to the neighbouring non-significant SNPs (marked with a green dashed line) to get the final signature of selection spanning the distance marked by a double arrow. [file 1471-2164-14-908-S3.pdf]
